# Patients with posttraumatic stress disorder exhibit an altered phenotype of regulatory T cells

**DOI:** 10.1186/1710-1492-10-43

**Published:** 2014-08-20

**Authors:** Mladen Jergović, Krešo Bendelja, Anđelko Vidović, Ana Savić, Valerija Vojvoda, Neda Aberle, Sabina Rabatić, Tanja Jovanovic, Ante Sabioncello

**Affiliations:** 1 Centre for research and knowledge transfer in biotechnology, University of Zagreb, Zagreb, Croatia; 2 Department of Psychiatry, University Hospital Dubrava, Zagreb, Croatia; 3 General hospital “dr. Josip Benčević”, Slavonski Brod, Croatia; 4 Department of Psychiatry & Behavioral Sciences, Emory University, Atlanta, GA, USA; 5 Department for Cellular Immunology, Institute of Immunology, Rockfellerova ulica 10, Zagreb, Croatia

**Keywords:** *Posttraumatic stress disorder*, *Regulatory T cells*, Autoimmunity

## Abstract

**Background:**

Regulatory T cells (Tregs) play a key role in immune homeostasis in vivo. Tregs have a critical role in preventing the development of autoimmune diseases and defects in Treg function are implicated in various autoimmune disorders. Individuals with *posttraumatic stress disorder* (*PTSD*) have higher prevalence of autoimmune disorders than the general population. We hypothesized that war veterans with PTSD would exhibit a decreased number and/or altered phenotype of Tregs.

**Methods:**

We analyzed peripheral blood mononuclear cells (PBMCs) of patients with PTSD (N = 21) (mean age = 45.9) and age-matched healthy controls (N = 23) (mean age = 45.7) to determine the proportion of Tregs and their phenotype according to the expression of CD127 and HLA-DR markers which describe the differentiation stages of Tregs. In addition, we analyzed the expression of membrane ectoenzyme CD39 on Tregs of the study groups, an important component of the suppressive machinery of Tregs.

**Results:**

We found no differences in the proportion of Tregs between PTSD patients and controls, but PTSD patients had a higher percentage of CD127^-^HLA-DR^-^ Tregs and a lower percentage of CD127^lo^HLA-DR^+^ Tregs compared to controls. There was no difference in expression of CD39 on Tregs of the study groups.

**Conclusions:**

Although the proportions of Tregs in PTSD patients were unchanged, we found that they exhibit a different phenotype of Tregs that might be less suppressive. Impaired differentiation and function of Tregs is likely involved in disruption of immune homeostasis in PTSD.

## Background

Regulatory T cells (Tregs) play a major role in maintaining homeostasis of the immune system by restraining the activity of effector cells and auto-reactive T lymphocytes, thus controlling immune reactivity. Tregs were first described in 1995 as a subset of CD4^+^ T cells expressing high levels of the interleukin (IL)-2 receptor alpha-chain (CD25) whose depletion led to development of autoimmune diseases [1]. Since CD25 is also expressed by antigen experienced and recently activated effector T cells, identification of transcription factor forkhead box P3 (FOXP3) as a specific intracellular marker of Tregs, has vastly improved the identification of this subpopulation of T cells [2,3]. X-chromosome encoded transcription factor FOXP3 is necessary for the development and function of Tregs and humans with mutation leading to loss of function in the FOXP3 gene develop a fatal lymphoproliferative autoimmune disorder, IPEX (immune dysregulation, polyendocrinopathy, enteropathy, X-linked) [4,5]. Although expressed predominantly by Tregs, FOXP3 is not an exclusive marker of Tregs [6] as it is induced during T-cell receptor (TCR) stimulation in conventional CD4^+^ T cells. Other markers such as low levels or lack of expression of IL-7 receptor CD127 have been suggested as useful in identifying peripheral Tregs [7].

Human T cells with regulatory function contain other FOXP3^-^ subpopulations (Th3, Tr1, iTr35, and CD8^+^CD28^-^) but CD4^+^CD25^hi^FOXP3^+^ are the most studied and comprehended in the context of homeostasis and autoimmunity in humans [8]. Studies of frequency of Tregs in autoimmune diseases such as rheumatoid arthritis, multiple sclerosis and psoriasis yielded conflicting results with systemic lupus erythematosus being the only autoimmune disease showing a consistent decrease of Tregs in the periphery [9]. However functional defects of Tregs have been observed in different human autoimmune diseases [10].

Expression of some surface receptors discriminates between functionally distinct Treg populations. HLA-DR is expressed on terminally differentiated Tregs which are more suppressive than the HLA-DR^-^ subset [11]. CD127 discriminates between human regulatory and activated T cells [12] and frequency of CD127 expressing cells within the FOXP3^+^ population correlates with poor Treg function [13]. CD39 is an ectoenzyme that degrades adenosine triphosphate (ATP) to adenosine monophosphate (AMP). It is expressed by some Tregs and conveys suppression by hydrolyzing extracellular ATP released by damaged cells [14]. CD39^+^ Tregs have the capability of suppressing IL-17 production and are impaired in multiple sclerosis [15].

*Posttraumatic stress disorder* (*PTSD*) is a trauma-related disorder that may develop after exposure to one or more traumatic events [16] and is characterized by recurring flashbacks, avoidance of memories of the event, emotional numbing, and hyperarousal. It is estimated that lifetime prevalence of PTSD in the general population is 6.8–8% [17,18] but combat veterans or individuals exposed to war have a higher risk of developing PTSD with reported prevalence rates ranging between 1.4% and 31% [19]. PTSD is associated with various biological and behavioral changes that constitute a higher risk for developing somatic illness [20] and higher mortality rates, especially from heart disease [21]. Several autoimmune or inflammatory disorders have been linked to PTSD, most notably rheumatoid arthritis [22], psoriasis and thyroid disease [23].

Studies of the immune system in PTSD have yielded conflicting results, with increased levels of pro-inflammatory cytokines (IL-1β, IL-6 and tumor necrosis factor (TNF)-α), lower total T cells counts, and increased reactivity to antigen skin tests being the most consistent findings [24,25]. Only one study has investigated Tregs in PTSD [26] finding decreased number of Tregs in blood of PTSD patients. Another study [27] showed a decreased number of Tregs in the peripheral blood of human subjects after an acute laboratory stressor. Studies in mice showed that chronic psychosocial stress induced a reduction of regulatory T cells in peripheral lymph nodes, accompanied by increased T cell effector functions [28].

We hypothesized that war veterans with PTSD would exhibit a decreased number and/or altered phenotype of Tregs. In addition to determining percentages of total Tregs (CD3^+^CD4^+^CD25^+^FOXP3^+^) in peripheral blood of PTSD patients, we further characterized Tregs according to their expression of HLA-DR, CD127 and CD39 to investigate whether PTSD patients exhibit a different phenotype of Tregs (possibly functionally distinct) compared to healthy controls.

## Methods

### Subjects

PTSD patients (n = 25) were Croatian male combat veterans, recruited among outpatients at General hospital “dr. Josip Benčević”, Slavonski Brod, Croatia who volunteered to participate in the study. All patients met the ICD-10 [29] PTSD criteria, the official classification in Croatian psychiatric practice. Healthy controls (n = 25) were men matched for age and demographics. The study was approved by the Ethic Committee of the hospital and written informed consent was obtained from all subjects.

Prior to phlebotomy, all study participants were examined by an experienced clinician (a psychiatrist), and medical history data relevant to the purpose of this study were recorded. The subjects were then asked to complete rating scales for PTSD, depression and anxiety symptoms.

The level of PTSD symptoms was assessed with the Los Angeles Symptom Checklist (LASC) [30] a self-report measure of PTSD and associated features. We used the 43-item version of the LASC to assess PTSD. Respondents rated the extent to which specific symptoms were a problem for them, using a 5-point scale ranging from 0 (*no problem*) to 4 (*extreme problem*). The diagnosis of PTSD was confirmed in all patients, i.e. out of 17 diagnostic items they reported (with a rating of four) at least one item assessing reexperiencing of the trauma, three items indexing avoidance and numbing, and two items reflecting increased arousal. Depression symptoms were assessed with the Beck Depression Inventory (BDI) [31] and current anxiety level (state anxiety) was determined by the corresponding part of Spielberger State-Trait Anxiety Inventory (STAI) [32].

Healthy controls did not meet lifetime or current criteria for any psychiatric disorder and had no symptoms or signs of current psychiatric disease. Due to ethical considerations the PTSD patients did not discontinue their regular medications. Standard laboratory tests were also performed in all participants. Two PTSD patients were excluded from the analysis because of high levels of CRP (>10 mg/L) indicating acute inflammation or infection. Another two PTSD patient were excluded because of chronic illness (multiple sclerosis and diabetes) and two controls were excluded due to very young age (more than 20 years below the group average). At the time of sampling, all other study subjects had no symptoms or signs of acute or chronic physical illness. None of the included participants reported intake of glucocorticoids.

Due to the ethical considerations, the majority of patients (n = 17) were medicated at the time of the study. They were taking selective serotonin reuptake inhibitors (SSRIs) (n = 12), benzodiazepines (n = 15), or antipsychotics (as an augmentation therapy, n = 8).

### Blood sampling and lymphocyte phenotyping

We collected fasting whole blood by venipuncture from 25 PTSD patients and 25 healthy controls on the same day between 7 and 9 AM. Blood was collected in sodium heparin treated tubes and serum tubes (BD Biosciences, Heidelberg, Germany). Serum tubes were sent to the hospital laboratory for measurement of C-reactive protein (CRP) levels to test for the presence of acute inflammation. Determination of CRP was performed using a standard latex immunoassay; CRP Vario (Abbott Diagnostics, Lake Forest, USA). Absolute numbers of lymphocytes were determined from whole blood using TruCOUNT™ Tubes (BD Biosciences). 50 μL of whole blood was incubated for 15 minutes with fluorescein isothiocyanate (FITC)-conjugated anti-CD45 (clone 2D1) and phycoerythrin (PE)-conjugated anti-CD14 (MφP9) (BD Biosciences) antibodies at room temperature, then treated with 450 μl of lysing buffer (BD Biosciences) for 10 minutes before flow cytometry was performed.

**Figure 1:**
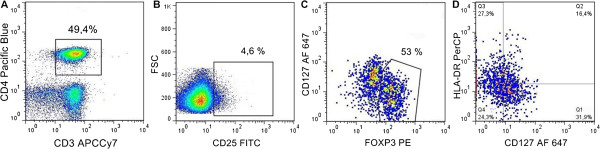
**Gating strategy for identification of Tregs populations.** Upon gating on CD3^+^CD4^+^ cells **(A)** a small percentage of cells with the highest expression of CD25 was gated **(B)** among these CD25^hi^ cells two distinct populations according to expression of FOXP3 and CD127 markers were visible **(C)**. Tregs were identified as FOXP3^+^CD127^lo/-^ subpopulation and divided into four subpopulations according to expression of HLA-DR and CD127 **(D)**.

Peripheral blood mononouclear cells (PBMCs) were isolated on Ficoll-Paque™ gradient (GE Healthcare Life Sciences, Uppsala, Sweden). Upon separation, mononuclear cells were washed, resuspended in freezing medium (10% FCS, 10% DMSO, 80% RPMI 1640) and transferred within a Freezing Container (Sigma-Aldrich, St. Louis, USA) to -80°C overnight, then stored in liquid nitrogen until further processing.. However, this was not a concern, since T cell subpopulations have been shown to be well preserved by cryopreservation [33]. Reports of cryopreservation effect on FOXP3 expression are conflicting, but the latest report indicates no effect if a medium with a low concentration of FCS is used [34]. We have tested the impact of the cryopreservation method by measuring Treg frequencies and phenotype in fresh and freeze/thawed PBMCs of a small random sample (N = 5). Frozen PBMCs were later quickly thawed in water bath at 37°C, resuspended in a pre-warmed RPMI 1640 medium and stained with antibodies for phenotypic analysis. For quantification of lymphocyte phenotypes, 500,000 cells were stained with Alexa Fluor^®^ 647 (AF 647)-conjugated anti-CD3 (HIT3a), Alexa Fluor^®^ 488 (AF 488)-conjugated anti-CD56 (HCD56), AF 488-conjugated anti-CD16 (3G8), PE-conjugated anti-CD19 (HIB19), peridinin-chlorophyll-protein (PerCP)-conjugated anti-CD8a (HIT8a), and Pacific Blue™ (PB)-conjugated anti-CD4 (RPA.T4) (Biolegend, San Diego, USA). Following antibody staining, samples were washed in a staining buffer and at least 20,000 cells were collected in the lymphocyte gate. Matching isotype controls were used for setting positivity gates: AF 647-conjugated mouse IgG2a, AF 488-conjugated mouse IgG1 kappa, PE-conjugated mouse IgG1 kappa, PerCP-conjugated mouse IgG1 kappa, PB-conjugated mouse IgG1 kappa (Biolegend).For quantification and phenotyping of Tregs 500,000 cells were stained with PB-conjugated anti-CD4, FITC-conjugated anti-CD25 (BC96), allophycocyanin (APC)/cyanine 7 (Cy7)-conjugated anti-CD3(HIT3a), PerCP-conjugated anti-HLA-DR (L243), AF 647-conjugated anti-CD127 (HCD127) (Biolegend) and PE-CY7-conjugated anti-CD39 (Ebioscience, San Diego, USA). After incubation in the dark for 15 minutes, cells were washed and fixed. The cells were then washed in a permeabilization buffer and stained with PE anti-human FOXP3 Staining Set (Ebioscience) following manufacturer instructions. Following antibody staining, samples were washed in the permeabilization buffer and at least 50,000 cells in the lymphocyte gate were collected. The isotype controls used for setting positivity gates were as follows: PB-conjugated mouse IgG1 kappa, FITC-conjugated mouse IgG1, kappa, APC/Cy7-conjugated mouse IgG2a kappa, PerCP-conjugated mouse IgG2a kappa, AF-conjugated 647 mouse IgG1 kappa (Biolegend) and PE-CY7-conjugated mouse IgG 1 kappa (Ebioscience). The gating strategy we used for identification and phenotypic characterization of Tregs is depicted in Figure 1. All samples were run on an LSRII flow cytometer (BD Biosciences) and data were analyzed with FlowJo software (Tree Star, Ashland, USA).

### Statistical analyses

Participants’ demographic characteristics with categorical data were compared using Fisher exact test for a 2 × 2 contingency table (vassarstats.net/tbl2x2.html). Distribution normality for all continuous variables was assessed per group by visual inspection of the data (shape and symmetry of distribution, outliers, P-P plot) and by Shapiro-Wilk’s *W*-test, performed with Statistica, v 6 (StatSoft Inc., Tulsa, USA). The effect of cryopreservation was assessed by comparing Treg frequencies and phenotype of the same subjects before and after cryopreservation with paired samples T test. Since the distribution of several variables was typically skewed, we used a nonparametric resampling procedure with 5,000 replications obtained from the complete study sample for all variables and tests applied. Descriptive statistics and effect sizes (Cohen’s d) with respective confidence intervals (CIs) were calculated from the original dataset using bootstrap (resampling with replacement) methods. Statistical inferences were verified by randomization (resampling without replacement) test. All sampling and computation was programmed using extended *Resampling Stats* language and run by Statistics 101, v 2.8 resampling statistics software (http://www.statistics101.net). Results are presented as means (an estimate of population mean based on 5,000 bootstrap samples) ± *SD* of the sampling distribution (the estimate of the sample *SE*) and effect sizes (ES, Cohen’s d) with associated 95% confidence intervals (CI). Two-tailed p-values were obtained as a proportion of the sampling distribution of absolute differences between 5,000 randomized group means (test statistic of interest) that are at least as extreme as the difference between original groups mean. Psychometric test scores (four LASC variables, one BDI and one STAI-S variable) and phenotype variables (nine variables) were correlated by using Spearman’s rank order correlations.

## Results

### Characteristics of participants

As shown in Table 1, the groups did not differ in respect of age, marital status, smoking habit and alcohol consumption. PTSD patients were slightly less educated, and a substantial proportion of this group was retired or unemployed. The patient group had higher scores in all psychometric tests with higher frequency and intensity of PTSD symptoms as well as higher levels of depression and anxiety symptoms. Although there are reports of low-grade inflammation determined by elevation of C-reactive protein in PTSD patients [35,36], we found no difference in CRP levels between groups (Table 1).

**Table 1:** Characteristics of participants

**Variables**	**PTSD patients (N = 21)**	**Healthy controls (N = 23)**	**p**
Age, years^a^	45.9 ± 1,02	45.7 ± 1.64	0.949
Education^b^			0.022
Elementary/high	21 (100)	17 (74)	
University	0 (0)	6 (26)	
Marital status^b^			0.335
Married	18 (86)	22 (96)	
Single/divorced	3 (14)	1 (4)	
Work status^b^			< 0.001
Employed	1 (5)	21 (91)	
Unemployed/retired	20 (95)	2 (9)	
Tobacco use^b^			0.081
Yes	14 (67)	9 (39)	
No	7 (33)	14 (61)	
Alcohol use^b^			0.125
Yes	15 (71)	21 (91)	
No	6 (29)	2 (9)	
CRP^a^, mg/L	2.5 ± 0.50	2.3 ± 0.39	0.822
BDI score^a^	30.3 ± 2.36	5.1 ± 1.18	< 0.001
LASC score^a^ re-experiencing	8.6 ± 0.47	1.3 ± 0.42	< 0.001
Avoidance	15.0 ± 1.05	4.4 ± 0.93	< 0.001
Arousal	22.5 ± 1.34	4.8 ± 1.00	< 0.001
17-item PTSD index	46.1 ± 2.63	10.4 ± 2.20	< 0.001
43-item full scale index	95.5 ± 5.48	36.0 ± 2.61	< 0.001
STAI-State^a^, score	49.0 ± 1.04	36.0 ± 2.61	0.001

^a^Data presented as mean (an estimate of population mean based on 5,000 bootstrap samples) ± *SD* of the sampling distribution (the estimate of the sample *SE*). The associated two-tailed p-values are obtained as proportion of sampling distribution of absolute differences between 5,000 randomized group means that are at least as extreme as the difference between original groups mean.

^b^Data presented as n (%). The associated p-values are Fisher’s exact two-tailed probabilities.

### Lymphocyte phenotyping

We have calculated and presented results of both hypothesis testing and the effect sizes of the difference in phenotypic variables between PTSD patients and healthy controls.

**Figure 2:**
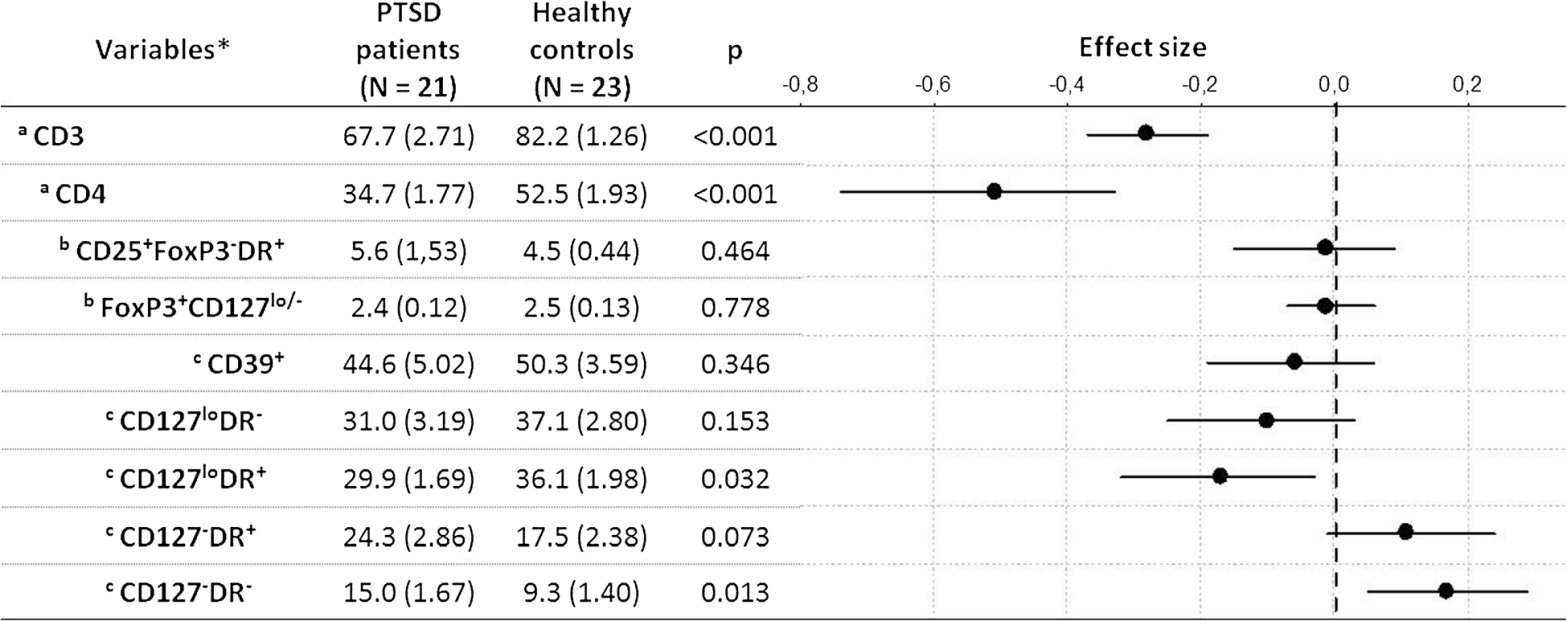
**Comparison of T cell subpopulations in PTSD patients and healthy controls.** Populations specified by indentation, beside stated markers, are characterized also by the first marker of all antecedent populations (e.g. full annotation for total Tregs, FoxP3^+^CD127^lo/-^, is CD3^+^CD4^+^CD25^+^FoxP3^+^CD127^lo/-^, see also Figure 1). Values are means of 5,000 bootstrap samples means (a point estimate of population mean) with SD of the sampling distribution (the estimate of the sample SE) in the parentheses. p-values are obtained as proportion of absolute differences between 5,000 randomized group means that are at least as extreme as the difference between original groups mean. In addition, effect sizes (dots) with associated 95% confidence intervals (horizontal bars) are depicted. *expressed as % of ^a^lymphocytes, ^b^CD4 lymphocytes, and ^c^total Tregs.

PTSD patients and healthy controls did not differ with respect to absolute numbers of lymphocytes (PTSD: 2444,4 ± 144,7 cells/μl; controls: 2719,8 ± 115,6, p = 0.13, ES = -0.11, 95% CI [-0.28, 0.03]), but PTSD patients had significantly higher proportions of cytotoxic T cells (CD3^+^CD8^+^) (PTSD: 27.5 ± 1.94%, controls: 20.9 ± 1.52%, p = 0.011; ES = 0.17 [0.05, 0.31]), B (CD3^-^CD19^+^) (PTSD: 5.7 ± 0.5%, controls: 2.7 ± 0.3%, p < 0.001; ES = 0.27 [0.18, 0.39] and NK cells (CD3^-^CD16^+^CD56^+^) (PTSD: 15.8 ± 2.5%, controls: 8.5 ± 0.7%, p < 0.01: ES = 0.15 [0.08, 0.22]). Results obtained for total T (CD3^+^), helper T (CD3^+^CD4^+^) cells, and their subpopulations, primarily Tregs subsets, are shown in Figure 2. PTSD patients had a significantly lower percentage of CD3^+^ (ES = -0.28 [-0.37, -0.19]) and CD3^+^CD4^+^ (ES = -0.51 [-0.74, -0.33]) cells compared to healthy controls. Upon gating on CD4^+^CD25^hi^ cells (Figure 1b), two populations were observed based on expression of FOXP3 and CD127 markers. We identified Tregs as a distinct population of CD3^+^CD4^+^CD25^+^FOXP3^+^CD127^lo/-^ cells (Figure 1c) and further analyzed expression of CD39 and HLA-DR markers on these cells (Figure 1d). As shown in Figure 2, there was no difference in the number of Tregs (ES = -0.01 [-0.07, 0.06]) but PTSD patients had a higher percentage of CD127^-^HLA-DR^-^ Tregs (ES = 0.17 [0.05, 0.29]) and a lower percentage of CD127^lo^HLA-DR^+^ Tregs (E = -0.17 [-0.32, -0.03]). As shown in Table 2. Cryopreservation method had no effect on Treg numbers and phenotype. Measured frequencies of Tregs in healthy controls and PTSD patients were similar or higher than in previous reports based on whole blood staining [26] further confirming no decrease in FOXP3 levels caused by cryopreservation. No difference in percentages of CD39^+^ Tregs (out of total Tregs) was observed between study groups (E = -0.06 [-0.19, 0.06]). In addition, we analyzed a subpopulation of recently activated non-regulatory helper T cells (CD4^+^CD25^+^FOXP3^-^HLA-DR^+^) (Figure 2) and found no difference between the study groups (E = -0.01 [-0.15, 0.09]).

**Table 2:** Effect of cryopreservation on Treg numbers and phenotype

	**Fresh PBMCs (N = 5)**	**Frozen PBMCs (N = 5)**	**p value**
CD3^+^CD4^+^	51,4	51,38	0,99
**Tregs** (CD3^+^CD4^+^CD25^+^FOXP3^+^CD127^lo/-^)	2,16	2,15	0,96
CD127^lo^HLA-DR^-^ Tregs	35,8	34,8	0,77
CD127^lo^HLA-DR^+^ Tregs	35,96	37,56	0,53
CD127^-^HLA-DR^+^ Tregs	20,54	20,8	0,91
CD127^-^HLA-DR^-^ Tregs	7,67	6,77	0,36

The impact of the cryopreservation method was tested by measuring Treg frequencies and phenotype in fresh and freeze/thawed PBMCs of a small random sample (N = 5). Treg frequencies and phenotype of the same subjects before and after cryopreservation were compared with paired samples T test (data represented as group means). The results indicate no effect of cryopreservation on Treg numbers and phenotype.

These results suggest that PTSD has a moderate effect (ES between 0.3 and 0.5) on total T and helper T cells and a small effect (ES between 0.1 and 0.2) on CD127^lo^HLA-DR^+^ and CD127^-^HLA-DR^-^ Tregs, but given that the associated CIs do not include zero, the effects may have important practical consequences.

### Correlations and subgroup analyses

BDI, STAI-State, and LASC (3 clusters of PTSD symptoms, 17-item PTSD index, 43-item full scale indeks) scores were correlated with phenotype variables in PTSD patients. Out of 54 correlations performed between phenotype variables and psychometric test scores, only two were statistically significant (without corrections for multiple comparisons) – 1) BDI vs. CD127^-^HLA-DR^-^ (Spearman R = -0.44, p = 0.045), and 2) LASC arousal vs. CD127^lo^HLA-DR^-^(R = 0.46, p = 0.033).

We have also compared all phenotype variables between patients who were taking SSRIs (n = 12), benzodiazepines (n = 15), or antipsychotics (n = 8) and those who were not using medication. The differences were tested using the same nonparametric resampling procedure as described in Statistical analyses section. All p values were above significance level (>0.05).

## Discussion

Although PTSD patients did not have decreased percentage of Tregs as hypothesized, our study shows an altered phenotype of Tregs in war veterans with PTSD. They had a higher percentage of CD127^-^HLA-DR^-^ Tregs and a lower percentage of CD127^lo^HLA-DR^+^ Tregs in comparison to healthy controls, suggesting a less suppressive phenotype that could increase susceptibility to autoimmune diseases.

We also observed some changes in major lymphocyte populations. The percentage of circulating T lymphocytes in PTSD patients was lower in comparison to healthy controls, which has been previously reported in men with a past history of PTSD [37]. However most studies showed no differences [38] or even higher numbers of T cells [39]. We also found a decreased proportion of circulating helper T cells as in previous studies [37,40] in contrast to other studies that have found no differences [41] or higher numbers of helper T cells [39]. These contradictory findings can be explained by the genetic and behavioral heterogeneity among PTSD patients and no conclusions on immune reactivity in PTSD patients should be drawn based on the sheer numbers of T cell populations. With this in mind our main goal was to examine the proportion and differentiation markers of Tregs in PTSD patients.

Only one previous study [26] examined Tregs in PTSD patients and reported a 48% reduction of regulatory T cells (CD4^+^CD25^hi^ FOXP3^+^) in chronic PTSD. We did not replicate the findings of this study as there was no difference in peripheral Tregs between our PTSD group and healthy controls. It is important to note that the inclusion of CD127 marker in our analysis allowed us a more accurate discrimination between Tregs and activated T cells. Most of the common markers used to characterize Tregs can also be expressed by activated non-regulatory T cells. Human FOXP3 was found to be transiently expressed in T cell receptor-stimulated CD4^+^CD25^-^ T cells that do not have regulatory properties [42]. CD127 is constitutively down-regulated on functional human Tregs, while it is highly expressed on effector/memory T cells although activated non-regulatory T cells have also been shown to down-regulate CD127 upon activation [43]. Thus we have identified the CD4^+^CD25^hi^FOXP3^+^CD127^lo/-^ subpopulation of T cells as the purest in Tregs but we cannot claim that absolutely all activated non-regulatory T cells were excluded from the analysis. Although there was no difference in Treg levels, we found that PTSD patients display a lower proportion of CD127^lo^HLA-DR^+^ Tregs and a higher proportion of CD127^-^HLA-DR^-^ Tregs. HLA-DR expression identifies a functionally distinct population of terminally differentiated human Tregs [11]. FOXP3 expression is significantly higher in the HLA-DR^+^ Tregs and these cells exhibit earlier kinetics of responder T cells (Tresp) suppression than the HLA-DR^-^ subset [10]. The most interesting difference in the Tregs phenotype was the percentage of CD127^-^HLA-DR^-^ cells which was significantly higher in PTSD patients (15%) than in healthy controls (9.3%). These cells are non-mature Tregs [44] that are less suppressive than HLA-DR expressing cells and were found to be impaired in multiple sclerosis patients. Thus it appears that PTSD patients might exhibit a less suppressive phenotype of peripheral Tregs although further ex vivo analysis of Tresp inhibition is needed to confirm this hypothesis. Studies of the immune system in PTSD have produced conflicting results although there is evidence for increased peripheral inflammation manifested by increased levels of pro-inflammatory cytokines (IL-1β, IL-6 and TNF-α) [24,25]. Since multiple mechanisms operate in Treg-mediated suppression, there are various ways through which altered Treg differentiation may contribute to the observed immune changes in PTSD. There are reports of both increased [26] and decresed [45] ex vivo proliferation of polyclonaly stimulated effector T cells in PTSD patients. Tregs suppress proliferation of other CD4+ and CD8+ T cells when Treg and responder populations are co-cultured and stimulated with specific antigen or a polyclonal TCR stimulator [46]. Therefore, to further examine functional consequences of the observed alterations in Treg phenotype, the distinct Treg subpopulations need to be isolated and cocultured with stimulated Tresp cells, which was beyond the scope of this study. Tregs also produce various cytokines such as IL-10, IL-4, IL-35 and IL-13 which are involved in the suppression of the pro-inflammatory cytokine response [46,47]. Additionally, Tregs can steer monocyte differentiation toward alternatively activated macrophages with strong anti-inflammatory potential [48]. Alterations in all of these Treg mechanisms of suppression could potentially contribute to the immunological abnormalities described in PTSD.

Future studies should establish whether the observed changes in the Treg phenotype are due to changes in differentiation and distribution of thymically derived FOXP3^+^ regulatory T cells (tTregs) or induction of peripheral Tregs (pTregs). This is complicated by the lack of specific markers that can distinguish tTregs from pTregs [8]. Thymically derived Tregs were shown to be able to convert to Th17 cells [49] in the presence of IL-6. Since higher levels of IL-6 in PTSD patients have been reported in several studies [24] Tregs conversion to inflammatory Th17 cells is an additional possible mechanism of immune dysregulation that needs to be further studied. Glucocorticoids inhibit the ability of antigen presenting cells to stimulate effector T lymphocytes and favor Tregs cell induction [50]. In a previous study our group has shown that lymphocytes isolated from PTSD patients exhibited decreased intracellular glucocorticoid receptor (GCR) levels compared with controls [51], thus signaling through GCRs is a way that neuroendocrine alterations in PTSD could affect Tregs differentiation and function. Defects of Tregs differentiation and function are likely involved in chronic inflammation implicated in PTSD and higher prevalence of autoimmune disorders observed in PTSD patients.

One of the limitations of the study is that we didn’t administer The Clinician-Administered PTSD Scale (CAPS), considered to be the “gold standard” in PTSD assessment [52]. Instead, a self-report scale (LASC) was used to assess the level of PTSD symptoms. We must emphasize that the PTSD group analyzed in this study consisted of treatment-resistant patients who started their treatment shortly after the war in Croatia. They were assessed on multiple occasions (yearly) with numerous tests including CAPS according to multimodal assessment principles [53]. The severity of their condition was also reflected in the high BDI scores (Table 1). One cannot exclude the possibility that the observed differences in cellular phenotype might have been the result of the comorbid depression in PTSD patients. However, out of all correlations performed between severity of depression symptoms (BDI scores) and phenotype variables, we found only one significant correlation - BDI scores negatively correlated with percentages of CD127^-^HLA-DR^-^ cells, i.e. patients with higher levels of depression symptoms tended to have lower percentages of Tregs with this phenotype. On the other hand, this subpopulation was found to be more prevalent in PTSD patients, indicating that PTSD, and not depression, was the more likely basis of this difference.

Another shortcoming of the study is that due to ethical considerations, the majority of patients were medicated at the time of the study. Although additional analyses did not reveal any significant associations of phenotype variables with the type of drug patients were using, this question remains open since the group sizes were too small to draw valid conclusions from these analyses.

## Conclusions

There is ample evidence in the literature for systemic inflammation and deleterious health consequences in PTSD, but the mechanisms of immune dysregulation in PTSD are not well understood. Results of this study indicate that altered differentiation of Tregs might be involved in the disruption of immune homeostasis in chronic stress. Since the prevalence of PTSD is fairly common in the general population and very high in individuals exposed to severe trauma, research on immune systems in PTSD is of great importance for understanding the mechanisms of trauma-related development of somatic disorders and developing future therapeutic interventions.

## Abbreviations

CRP: C-reactive protein; BDI: Beck Depression Inventory; GCR: Glucocorticoid receptors; LASC: Los Angeles Symptom Checklist; PBMCs: Peripheral blood mononouclear cells; *PTSD*: *Posttraumatic stress disorder*; STAI: State-Trait Anxiety Inventory; Tregs: Regulatory T cells; Tresp: Responder T cells.

## Competing interests

The authors declare that they have no competing interests.

## Authors’ contributions

MJ performed laboratory experiments, analysis of flow cytometry data and wrote the first draft of the manuscript. KB, AV, TJ and SR conceived and designed the experiments. AS and VV isolated leukocytes and performed the experiments. AS performed statistical analyses of the data and contributed to the study design. NA organized and conducted psychological testing and blood sampling. All authors read and approved the final manuscript.
